# Successful Implementation of Expanded Newborn Screening in the Philippines Using Tandem Mass Spectrometry

**DOI:** 10.3390/ijns8010008

**Published:** 2022-01-19

**Authors:** Carmencita D. Padilla, Bradford L. Therrell, Maria Melanie Liberty B. Alcausin, Mary Anne D. Chiong, Mary Ann R. Abacan, Ma. Elouisa L. Reyes, Charity M. Jomento, Maria Truda T. Dizon-Escoreal, Margarita Aziza E. Canlas, Michelle E. Abadingo, J. Edgar Winston C. Posecion, Conchita G. Abarquez, Alma P. Andal, Anna Lea G. Elizaga, Bernadette C. Halili-Mendoza, Maria Paz Virginia K. Otayza, David S. Millington

**Affiliations:** 1 Newborn Screening Reference Center, National Institutes of Health, University of the Philippines Manila, Manila 1000, Philippines; mbalcausin@up.edu.ph (M.M.L.B.A.); mlreyes9@up.edu.ph (M.E.L.R.); cmjomento@up.edu.ph (C.M.J.); mdescoreal@up.edu.ph (M.T.T.D.-E.); mecanlas@up.edu.ph (M.A.E.C.); meabadingo@up.edu.ph (M.E.A.); 2 Department of Pediatrics, College of Medicine, University of the Philippines Manila, Manila 1000, Philippines; mdchiong1@up.edu.ph (M.A.D.C.); mrabacan@up.edu.ph (M.A.R.A.); 3 Institute of Human Genetics, National Institutes of Health, University of the Philippines Manila, Manila 1000, Philippines; 4 National Newborn Screening and Global Resource Center, Austin, TX 78759, USA; therrell@uthscsa.edu; 5 Department of Pediatrics, University of Texas Health Science Center at San Antonio, San Antonio, TX 78229, USA; 6 Department of Biochemistry, Molecular Biology and Nutrition, Faculty of Medicine and Surgery, University of Santo Tomas, Manila 1008, Philippines; 7 Newborn Screening Center—Visayas, West Visayas State University Medical Center, Iloilo 5000, Philippines; eposecion@gmail.com; 8 Newborn Screening Center—Mindanao, Southern Philippine Medical Center, Davao 8000, Philippines; conch.abarquez@gmail.com; 9 Newborn Screening Center—Southern Luzon, Daniel O. Mercado Medical Center, Tanauan 4232, Philippines; ampanganiban@dmmcinc.com; 10 Newborn Screening Center—National Institutes of Health, Quezon 1101, Philippines; agelizaga@up.edu.ph; 11 Newborn Screening Center—Central Luzon, Angeles University Foundation Medical Center, Angeles 2009, Philippines; bernadettehmendozansc@gmail.com; 12 Newborn Screening Center—Northern Luzon, Mariano Marcos Memorial Hospital and Medical Center, Batac 2906, Philippines; bingotayza@gmail.com; 13 Department of Pediatrics, Duke University School of Medicine, Durham, NC 27708, USA; milli014@duke.edu

**Keywords:** expanded newborn screening, tandem mass spectrometry, Philippines, metabolic screening

## Abstract

Newborn bloodspot screening (NBS) began as a research project in the Philippines in 1996 and was mandated by law in 2004. The program initially included screening for five conditions, with a sixth added in 2012. As screening technology and medical knowledge have advanced, NBS programs in countries with developed economies have also expanded, not only in the number of newborns screened but also in the number of conditions included in the screening. Various approaches have been taken regarding selection of conditions to be screened. With limited resources, low- and middle-income countries face significant challenges in selecting conditions for screening and in implementing sustainable screening programs. Building on expansion experiences in the U.S. and data from California on Filipinos born and screened there, the Philippine NBS program has recently completed its expansion to include 29 screening conditions. This report focuses on those conditions detectable through tandem mass spectrometry. Expanded screening was implemented in a stepwise fashion across the seven newborn screening laboratories in the Philippines. A university-based biochemical genetics laboratory provides confirmatory testing. Follow-up care for confirmed cases is monitored and provided through the NBS continuity clinics across the archipelago. Pre-COVID-19 pandemic, the coverage was 91.6% but dropped to 80.4% by the end of 2020 due to closure of borders between cities, provinces, and islands.

## 1. Introduction

Newborn bloodspot screening (NBS) is a successful public health prevention system that has evolved over the sixty years since the initial work of Guthrie in the U.S. [1]. While initially focused on a single congenital metabolic condition resulting in mental retardation, phenylketonuria (PKU), NBS now includes varying numbers of additional conditions that can result in serious outcomes, including death, when not detected and treated early. The goal of NBS remains focused on diminishing morbidity and mortality and is generally acknowledged as consisting of a six-part system that includes education, screening, short-term follow-up, diagnosis, treatment/management (long-term follow-up), and evaluation [2]. Increased disease knowledge, including natural history and treatment, and improved analytical techniques have resulted in the inclusion of increasingly larger numbers of conditions on screening panels in NBS systems in countries with high-income economies and smaller numbers in lower middle-income countries (LMIC) [3].

The Philippines, which is a LMIC, faces special challenges as an archipelago of over 7600 islands and 110 ethnolinguistic groups, and currently with 110 M population and annual births of 1.8 M. The Newborn Screening Study Group, consisting of pediatricians and obstetricians from 24 hospitals, initiated the Philippine Newborn Screening Project (PNSP) in 1996 [4,5,6]. The prevalence of five screening conditions—congenital hypothyroidism (CH), congenital adrenal hyperplasia (CAH), galactosemia (GAL), phenylketonuria (PKU), and homocystinuria (HCY), supported the adoption of NBS across the Philippines [5]. NBS coverage gradually expanded across the country until today, where over 7400 newborn screening facilities (NSFs), i.e., birthing centers, submit NBS screening specimens covering over 90% of all Philippine newborns [7].

Pilot screening for glucose-6-phosphate dehydrogenase (G6PD) deficiency in 1998, revealed a significant incidence [8]. It replaced HCY on the NBS panel in 2000 when HCY was removed due to a lack of case finding. With the support of the Department of Health [9], the President of the Republic [10], and a 2004 Congressional mandate to “*ensure that every baby born in the Philippines is offered the opportunity to undergo newborn screening,*” [11] NBS moved forward. In 2006, NBS was included as a national health benefit by the Philippine Health Insurance Corporation (PHIC), the national health insurer [12]. This action resulted in a significant uptake of the program since the out-of-pocket screening fee was eventually eliminated. The only other screening condition added to the Philippine NBS panel prior to serious considerations about expanded NBS (ENBS) was maple syrup urine disease (MSUD) [13]. MSUD was added in 2012 as a result of relatively large numbers of clinical cases in the pediatric population and discovery of a novel mutation in the Philippines [14].

During the time period from 1996–2012, as the number of newborns screened and cases detected through screening increased, the importance of quality NBS services also increased. Administrative and functional infrastructure quality improvements were continually evaluated and improvements implemented. While the 2004 NBS law assigned program implementation to the Department of Health (DOH) through the National Technical Working Group (NTWG) [15], a significant and essential collaboration existed with the National Institutes of Health (NIH), University of the Philippines Manila. The Newborn Screening Reference Center (NSRC) within the NIH serves as the Secretariat of the Advisory Committee on NBS and the technical arm of the NBS program. As such, it serves an active role in: (1) defining testing and follow-up protocols; (2) maintaining an external laboratory proficiency testing program; (3) overseeing the national testing database and case registries; (4) assisting in training activities across the program; (5) overseeing the content of educational materials; and (6) recommending the establishment of newborn screening centers (NSCs) [15]. Its critical role in assuring quality screening performance nationally is essential and the methodologies employed have been recently published [16].

Also, during this time period, disease natural histories, analytical micro-techniques, and clinical management/treatments were advancing globally. Specific and relevant NBS advancements included DNA screening techniques for both hemoglobinopathies (HGB) and cystic fibrosis (CF), and tandem mass spectrometry (MS/MS) for inborn errors of metabolism. The evolution of NBS in the 1960s had included comprehensive discussions and debates on what, when, and how additional conditions should be included on screening panels, [2] and most NBS programs focused their screening panel selections on Wilson and Jungner’s principles of population screening utilizing a single screening test for each condition screened [17]. Multiplex testing in the 1990s, which allowed several conditions to be simultaneously detected from a single specimen, began to significantly affect the decision-making matrix governing screening panel disorders, first in the U.S. [18,19,20] and later in Europe [21,22]. A group of reports outlining the status of NBS worldwide was published in 2007 showing widespread NBS program expansions internationally in response to rapidly advancing knowledge and technical capabilities [23,24,25,26,27]. Additionally, NBS programs in many different countries, including some in Asia, were reporting on the successes of multiplex MS/MS in expanding the metabolic conditions that could be tested [28,29,30,31]. (**Note:** In reviewing numbers of conditions screened, it is important to note that the procedure for counting conditions is not yet harmonized, resulting in variable tabulations of numbers of conditions included on NBS screening panels [32]).

NBS expansion, while extremely important in reducing newborn morbidity and mortality, cannot be successfully implemented without careful evaluation and planning, particularly in a LMIC setting. This report describes the process used by the Philippine NBS program for considering which conditions to include in ENBS, a simplified method for gathering pilot data, and implementation methodology for certain metabolic conditions screened by MS/MS. It is intended to provide information that might be helpful for LMICs facing similar conditions.

## 2. Methodology

Completion of pilot studies to prove the value of adding a condition or group of conditions to a NBS program can be both time-consuming and expensive. Thus, the feasibility of obtaining useful data on conditions screened, methodologies used, and costs incurred from another NBS program surveying a similar population of newborns may present a valid alternative to pilot testing, depending on the characteristics of the screened population. We determined that the California Newborn Screening Program (CNSP), which is similarly organized to the Philippine NBS Program and at the time included screening for more than 70 different conditions, likely would provide sufficient Filipino NBS data to aid in evaluating other congenital conditions suitable for inclusion in the Philippine NBS Program. Specimen collection requirements of the CNSP are similar to those in the Philippine NBS program, with seven state-approved laboratories performing the screening tests and a similarly sized network of follow-up clinics. We contacted the CNSP and were able to obtain data on Filipino newborns both for conditions included in the Philippine NBS program and for others not yet included. In addition to data on the six NBS tests available at that time in the Philippines and HGB (reported separately [7]), other conditions on the US Recommended Uniform Screening Panel (RUSP) [19,20], including certain fatty acid oxidation (FAO) disorders, amino acid (AA) disorders, and organic acid (OA) disorders, screened using MS/MS were included. Data for biotinidase deficiency (BIO) and cystic fibrosis (CF) were also included. Once obtained, the CNSP data were analyzed and extrapolated to the entire Philippine newborn population and presented to the NTWG to assess the value of expanding the Philippine NBS Program. In order to better evaluate costs and cost effectiveness, we also carefully reviewed pertinent published costing studies with particular attention to those focusing on MS/MS [33,34,35] and studies from the CNSP [36].

At the time expansion was being considered, the Philippine NBS Program had essentially no technical expertise in advanced NBS micro-analytical methods such as MS/MS and there was limited commercial product support for complex analytical instruments not manufactured locally. For these reasons, and because MS/MS appeared to be the primary screening methodology for the additional metabolic conditions [37,38], major emphasis was placed on understanding and evaluating MS/MS as a NBS tool. Intensive discussions with international experts experienced in MS/MS NBS provided major input into evaluation, planning, and strategies regarding potential challenges of NBS program expansion. We also reviewed published experiences from other NBS programs that assessed the strengths and weaknesses of various brands of screening equipment, laboratory workflow processes, and reporting/tracking/follow-up protocols.

**Figure 1:**
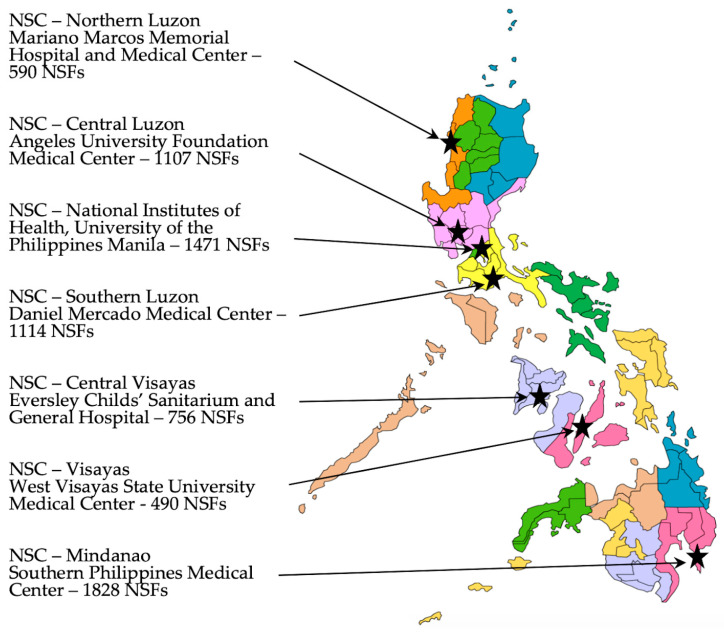
Location map of the seven Newborn Screening Centers (NSCs) currently providing screening laboratory services along with the number of Newborn Screening Facilities (NSFs) served by each. The seventeen different government regions are illustrated by the different colors.

To begin efforts to develop knowledge of metabolic disease detection using MS/MS, educational workshops for Philippine stakeholders were organized. We became aware of MS/MS training workshops ongoing in the US and obtained information from the US National Newborn Screening and Global Resource Center (NNSGRC). Rather than send students to the US training courses, we invited one of the principal MS/MS trainers and a NNSGRC representative to conduct an introductory workshop in the Philippines focused on implementing an expanded NBS program for metabolic disorders. This first workshop was attended by local geneticists, neonatologists, follow-up nurses, NSC directors, and laboratory managers (at the time, there were six NSCs in the country (now seven)—see Figure 1), and other program support staff (including selected administrative, laboratory, follow-up, and quality assurance personnel). Faculty for this three-day workshop included the two invited international experts in NBS and MS/MS, a local metabolic specialist/program consultant, and local NBS program administrators. Workshop content included medical information on various metabolic conditions detectable by MS/MS, algorithms for their detection (including post screening laboratory tests), international NBS experiences with MS/MS case detection and follow-up, and potential challenges in implementing MS/MS testing in the Philippines. Once trained, Philippine NBS personnel provided additional workshops to nursing staff.

As policy deliberations continued regarding which conditions to add to ENBS, instrument manufacturers were contacted to provide their input into equipment availability and related subjects (service, parts, etc.). A second workshop was planned to provide further training and review using the same invited experts as the first workshop. Additionally, a visit to the US training facility providing NBS MS/MS training was arranged for the local metabolic expert. Progress in obtaining MS/MS equipment through local ordering processes proceeded and an equipment contract was awarded that included onsite training by a different MS/MS expert.

Subsequently, additional workshops were conducted by the MS/MS instrument manufacturer oriented towards instrument operation but including other information about individual disease detection protocols. These workshops targeted both instrument operators in the various NSCs and others assisting in follow-up. Conference calls with the international experts associated with all workshops were conducted as issues requiring their input or clarification arose. In addition to the orientation visit of the local metabolic specialist to the US training facility, the Philippine NBS Quality Assurance Officer also attended one of the MS/MS training courses in the US. An education and information sharing plan was developed to inform physicians, parents, and other stakeholders, including the national insurance provider, about program expansion. A phased-in laboratory approach was also planned, which included consideration of other conditions being simultaneously added to the screening panel and targeted for implementation at about the same time.

## 3. Results

NBS data for 111,127 Filipino newborns born and screened in California, USA between 7 July 2005 and 6 July 2011 were obtained from the CNSP and analyzed (see Table 1) [39,40]. Included were all conditions on the US Recommended Uniform Screening Panel (RUSP) except hearing screening. The data for BIO and CF were limited to newborns screened after 16 June 2007 (3 years of data versus 6 years for all others). An analysis of the CNSP data (Table 2) was completed, presented to the NTWG, and the proposal to move forward with the inclusion of an additional 19 metabolic conditions, CF, BIO, and HGB was accepted. A National Technical Working Group for Expanded Newborn Screening (NTWG-ENBS) was created in response to the urgency and importance of implementation [41]. The NTWG-ENBS included DOH staff and representatives from other stakeholder institutions. It was subsequently divided into several smaller committees that were assigned responsibilities for:

1. Operations—develop operational infrastructure (fees, laboratory expansion, and follow-up).
2. Outreach—prepare guidelines for medical centers/personnel involved in follow-up.
3. Advocacy—preparing/distributing promotional/educational/training materials announcing/clarifying expansion.

Two working groups under the DOH Family Health Office, Disease Prevention and Control Bureau developed medium- and long-term goals for the ENBS program. The DOH established enabling rules through an administrative order (AO) that clearly defined preparations needed before implementation of ENBS and operational parameters for its implementation, including the fee structure. Additionally, an in-depth review of the capabilities of each NSC was made using the AO as its basis.

A single NSC, the NSC-National Institutes of Health (NIH) in Quezon City, was chosen to order and install equipment, add necessary personnel, and begin screening; this process took approximately 18 months. The MS/MS instrument supplier provided careful training throughout the installation process including proficiency evaluation materials and hands-on training. The Newborn Screening Quality Assurance Program (NSQAP) of the Centers for Disease Control and Prevention (CDC), USA, helped to ensure the quality of laboratory testing by providing limited quality control (QC) and proficiency testing (PT) specimens to assist with laboratory implementation. Successful analysis of both manufacturer’s and NSQAP materials were essential in building confidence in procedures and assuring quality results. Subsequently, the Quality Assurance (QA) Officer of the NSRC coordinated with the NSQAP to include the seven NSCs in their PT program. The long-term plan is for the NSRC to prepare QC materials for external PT of the NSCs. A standardized internal QC is followed at each NSC laboratory.

Once the equipment was validated and personnel appropriately trained, increasing numbers of proficiency specimens were tested. The courier system existing between newborn screening facilities (NSFs) and NSCs was reviewed by the program’s quality assurance officer and shown to provide timely specimen transport and environmental safeguards sufficient to prevent specimen damage due to time or heat/moisture during transport. Final validation of laboratory testing protocols included satisfactory analysis of 2250 specimens invited from 42 NSFs over a 15-day period in July 2014. Because ENBS was not a covered benefit of the PHIC program at the time, parents were required to pay a small fee for the additional testing. Specimens were officially accepted for ENBS beginning in December 2014 at NSC-NIH and 13 specimens were screened by the end of the year.

Training continued using NSC-NIH as the center and by collaborating with the MS/MS instrument supplier to attain sufficient testing proficiency for screening implementation at the various NSCs. As training was accomplished, screening was integrated into the screening activities of NSC-Visayas, Iloilo City in November 2015; NSC-Central Luzon, Angeles City, Pampanga in January, 2016; NSC-Mindanao, Davao City in July 2017; NSC-Southern Luzon, Tanauan City, Batangas in July, 2018; NSC-Northern Luzon in Batac City, Ilocos Norte in January 2019; and NSC-Central Visayas in Mandaue City, Cebu in February 2020 (locations shown in Figure 1). As ENBS was officially added to each NSC’s laboratory activities, that laboratory was enrolled in the external proficiency testing program at NSQAP and their results evaluated as part of the DOH certification program.

An Experts’ Committee on ENBS, knowledgeable in the disorders on the screening panel, was organized to provide new information on the disorders, participate in the review of datasets and cutoffs for the disorders and outcomes, and to propose research questions and recommend inclusion of new disorders. The Biochemical Genetics Unit of the Institute of Human Genetics, National Institutes of Health University of the Philippines Manila (IHG-NIH), provides reference services for specimens with initial out-of-range MS/MS results. Mutational testing for fatty acid oxidation disorders is sent to Invitae, San Francisco, CA, USA.

## 4. Discussion

NBS is the most successful genetic screening program in the Philippines. Its successes over time can be directly linked to its inclusion in the public health system. Table 3 summarizes the official actions that contributed (and continue to contribute) to its institutionalization. It was integrated by law into the public health delivery system as the National Comprehensive NBS System (NCNBSS) in 2004. This law and its enabling rules ensured that: (1) every baby born in the Philippines is offered NBS; (2) a sustainable NBS system exists and is integrated into the public health delivery system; (3) all health practitioners are aware of the benefits of NBS and of their responsibilities to offer it; and (4) all parents are aware of NBS and their responsibility to protect their child from any of the included disorder [42]. ENBS increased the Philippine newborn screening panel from six to twenty-eight conditions, including hemoglobinopathies [7], selected amino acid, organic acid, and fatty acid oxidation disorders, CF, and BIO. Two DOH AOs addressed implementation of ENBS in some detail [41,43]. Once ENBS began at the end of 2014, persistent case findings of argininosuccinic aciduria (ASA) when resolving some of the MS/MS results led to its inclusion on the Philippine NBS panel in 2018 increasing the total number of screened disorders to 29.

Several challenges to MS/MS implementation were encountered during the 3-year preparation period including: (1) forecasted space requirements for shifting from six tests to ENBS were inadequate for some NSCs (approval of insurance coverage for ENBS drastically increased demand, which increased the need for laboratory supplies, storage, and work space); (2) reconfiguration of the laboratory information management system (LIMS) was tedious; and (3) differences in laboratory practices between the NSCs were observed during external audits and accreditation reviews. These problems were addressed through inclusion of adequate laboratory workspace and staffing estimates as requirement for renewal of accreditation, closer coordination between the NSRC, the NSCs and the LIMS provider, and development of standard laboratory manuals for all procedures, respectively.

**Figure 2:**
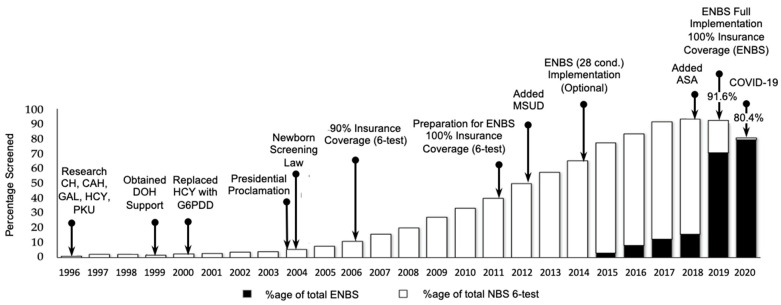
Timeline and screening coverage for various stages of implementation of NBS in the Philippines. National insurance coverage for ENBS was approved in 2018. Despite the COVID-19 pandemic, which began in early 2020, NBS coverage in 2020 exceeded 80% and ENBS was 79.4%. Abbreviations: CH = Congenital Hypothyroidism; CAH = Congenital Adrenal Hyperplasia; GAL = Galactosemia; HCY = Homocystinuria; PKU = Phenylketonuria; DOH = Department of Health; G6PDD = Glucose-6-Phosphate Dehydrogenase Deficiency; MSUD = Maple Syrup Urine Disease; ASA = Argininosuccinic Aciduria; ENBS = Expanded Newborn Bloodspot Screening.

Although the cost of screening for the 6-test panel was a fully-covered benefit of PHIC beginning in 2006, screening for the extra ENBS conditions was not covered when testing began. A fee was necessary whenever ENBS was requested. As the benefit data became clearer, PHIC was able to include full coverage of ENBS beginning in 2018 [44]. As a result, ENBS coverage increased from 2.8% in 2015, to 50.5% in 2018, and 70.2% in 2019 (see extended timeline in Figure 2). The overall NBS coverage (ENBS + six-test panel) was 91.6% at the end of 2019 and dropped slightly to 80.4% at the end of 2020 as a result of the COVID-19 pandemic due to closure of borders between cities, provinces, and islands. The fact that the number of specimens received and tested only decreased slightly is a credit to the various teams within the NBS program (screening facilities, screening laboratories, and follow-up) that have remained operational throughout the pandemic. Each has responded as needed to various challenges including specimen collection, specimen transport across closed provincial borders, staff shortages due to illness, and management of screened positive patients. Telemedicine was routinely utilized as part of patient follow-up. Other government agencies also willingly assisted with the delivery of NBS services including Local Government Units, the Philippine Air Force, the Office of Civil Defense, and the Philippine National Police, among others.

Timely and effective medical management is essential for successful NBS and a systems approach that addresses case detection, referral, treatment, and long-term follow-up is required for maximum effectiveness [45]. In order to monitor and provide more timely and comprehensive follow-up services, including case management assistance and testing support for indigents, 15 NBS Continuity Clinics (NBSCCs) have been established and strategically located across the 17 government regions [46]. Once diagnosis of a condition is made, the patient is endorsed to the continuity clinic for long-term follow-up care. Depending on available funds, the long-term goal is to create NBSCCs at the provincial level. In addition to a physician and a nurse, NBSCC medical follow-up teams are intended to include a genetic counselor, where possible. Currently, the number of genetic counselors in the Philippines is severely limited. A Master’s degree program for genetic counselors has been initiated at the University of the Philippines Manila in an attempt to better meet this need [47].

**Table 3:** Official actions contributing to the successful implementation of Philippine Newborn Screening Program and Expanded Newborn Screening (adapted from reference [48]).

No.	Action	Title (Description)
1	AO No. 1-A 2000	Policies on the Nationwide Implementation of NBS
2	Dept. Order No. 29-C s 2001	Creation of the NTWG on NBS Program
3	AO No. 121 s 2003	Strengthening Implementation of the NBS System
4	DM No. 59 s 2004	Establishment of the Accreditation of NSCs
5	Presidential Proclamation No. 540 (20 January 2004)	Declaring the First Week of October of each year as “National Newborn Screening Awareness Week”
6	Republic Act 9288 or Newborn Screening Act of 2004	An act promulgating a comprehensive and national system for ensuring NBS
7	Implementing Rules and Regulations for RA 9288	Promulgates the implementation of RA 9288
8	AO No. 2005-005	Cost of the NBS and Maximum Allowable Service Fees for the collection of NBS samples in all NSCHF
9	DM No. 2007-108	Ensuring that all newborns shall have access to NBS
10	AO No. 2007-0027	Revised Rules and Regulations Governing the Licensure and Regulation of Clinical Lab in the Philippines
11	DM 2008-0020	Reiterating the Provision of NBS Services as a Mandatory Licensing Requirement for all Hospitals
12	DM No. 2008-0114	G6PD Confirmatory Laboratories
13	AO No. 2008-0029	Implementing Health Reforms for Rapid Reduction of Maternal and Neonatal Mortality
14	DM No. 2009-0025	Hiring of Full-time Staff Coordinators for the NBS Program
15	AO No. 2009-0025	Adopting New Policies and Protocol on Essential Newborn Care
16	AO No. 2009-0028	Designation of the NSRC, NIH-UPM to Oversee the Quality Assurance Program for G6PD Test
17	AO No. 2012-0017	Dried Blood Spots Guidelines
18	AO No. 2012-0154	Inclusion of MSUD in the NBS Panel of Disorders
19	AO No. 2013-0015	Guidelines on the NBS DOH CHD and ARMM 4% Fund Utilization
20	AO No. 2014-0035	Implementing Guidelines on the Setting-up of NBS Continuity Clinics
21	AO No. 2014-0045	Guidelines on the Implementation of the Expanded NBS Program
22	AO No. 2018-0025	National Policy and Strategic Framework on ENBS from 2017–2030
23	AO No. 2020-0052	Revised Guidelines on the Implementation on the ENBS Program

Abbreviations: AO = Department of Health Administrative Order; Dept. = Department of Health; DM = Department of Health Memorandum; NBS = Newborn Bloodspot Screening; NTWG = Newborn Screening Technical Working Group; NSC = Newborn Screening Center; RA = Republic Act; NSCHF = Newborn Screening Collecting Health Facilities; G6PD = Glucose-6-Phosphate Dehydrogenase; MSUD = Maple Syrup Urine Disease; DOH = Department of Health; CHD = Center for Health Development; ARMM = Autonomous Region in Muslim Mindanao; ENBS = Expanded Newborn Screening.

When ENBS became fully covered by national health insurance, the increased newborn coverage required concurrent expansion of the NBS follow-up system. Three Centers for Human Genetic Services (CHGS) were established, with administrative and operational oversight from the NIH-IHG, to provide clinical oversight and consultative services for the NBSCCs. These CHGSs were established to safeguard the continuity and sustainability of quality testing, follow-up services, and clinical management of diagnosed newborns. Each CHGS is staffed with a clinical geneticist (and other medical professionals defined in the enabling administrative order [43]) who assist with linking primary care physicians to regional disease specialists for the NBS panel of diseases. The CHGS also assist with data accumulation, review, and evaluation of long-term outcomes. The NIH-IHG serves as the CHGS for Luzon, with satellite CHGSs located in the Visayas and Mindanao. The CHGS network is expected to expand to Northern Luzon and Northern Mindanao as funds and staff are available.

**Figure 3:**
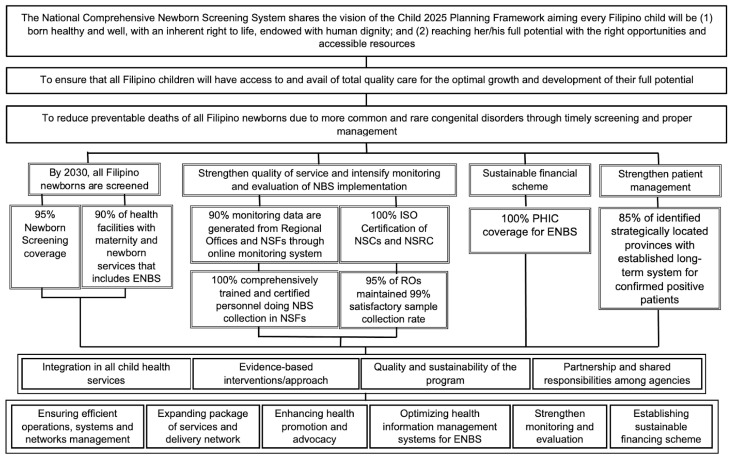
Planning diagram (2017—2030) for expanded newborn screening (adapted from reference [48]). Abbreviations used: NBS = Newborn Bloodspot Screening; ENBS = Expanded Newborn Bloodspot Screening; ISO = International Organization for Standardization; NSC = Newborn Screening Center; NSF = Newborn Screening Facility; PHIC = Philippine Health Insurance Corporation; RO = Regional Office.

ENBS also brought with it the need to continue planning for future program improvements and possible expansions. The DOH-defined NBS program objectives for 2030 focus on ensuring that all Filipino newborns are screened, program quality is strengthened, program operations and patient management are monitored and evaluated, and a sustainable financing scheme exists. Figure 3 is part of a comprehensive AO from the DOH that forms the basis for ENBS plans and development ideas for the 2017–2030 time period [48] and is presented here as a template that might be useful for other expanding programs. In addition to the vision, mission, and goals given in the first three boxes, program objectives, targets in support of the objectives, guiding principles, and strategies for successful implementation are also listed.

## 5. Conclusions

The stepwise implementation of ENBS in the Philippines has resulted in successful program expansion, despite the challenges of a LMIC environment. The added expenses of pilot screening to consider inclusion of additional disorders were avoided by obtaining and analyzing expanded screening data from California, USA on a representative population of Philippine newborns. Collaboration with the NNSGRC and Duke University Medical School in Durham, North Carolina, USA provided direction and training in the new screening technique of MS/MS, and commercial product vendors assisted with instrument installation, screening practice, and additional staff training. Inclusion of ENBS in the Newborn Care Package of PHIC was essential for increasing screening availability to the general population. Despite the COVID-19 pandemic, there was an increase of ENBS samples in 2020 and the same appears to be true for 2021. While laws and other official actions may not be necessary for successful screening in some jurisdictions, their consideration and implementation in the Philippines has had a positive effect on the institutionalization and implementation of ENBS. The intersection with public health was (and is) essential to the success of a national newborn screening program.

Through our Experts’ Committee on ENBS, we continue to review cases, finding data in the Philippines and elsewhere that might provide information on other disorders that should be included in ENBS. Similarly, we strive to keep abreast of changes in screening approaches globally. For example, we are monitoring changes in the approach to disorder selection in Japan [29], in the U.S. [49], which now focuses more on evidence and system readiness, and in Europe [21,22,50] and Australia [51], where considerations are aimed at program harmonization across jurisdictions. We have also developed educational information for physicians building on the American College of Genetics and Genomics (ACMG) ACT Sheets [52] and other needs identified for family and emergency room physicians [53,54].

Implementation of ENBS poses challenges in a LMIC environment. The success of expansion plans to ENBS depends on the close coordination of the ENBS implementers, Department of Health and Local Government Units, insurance providers, hospitals and birthing centers, health professionals (physicians, nurses, and midwives), and parents. The major steps used by the Philippine program in the implementation of ENBS were: (1) review of literature and established ENBS programs to review challenges in the way of expansion to ENBS; (2) review of local cases diagnosed through genetic clinics to convince policy makers of the value of ENBS to the newborns, families, and society; (3) conduct dialogues with public health officials and the national insurance provider on NBS expansion plans; (4) conduct educational workshops led by international experts (laboratory and clinical); (5) conduct training exercises for local implementers (laboratory staff, specialists, and administrative staff); (6) development of standard laboratory manuals and clinical guidelines; (7) preparation of short-term and long-term follow-up clinics for the patients diagnosed by the program; (8) development of policies for implementation of ENBS; (9) phased-in implementation of ENBS; and (10) continuous evaluation and monitoring and improvement of the program. Collaborations with other more advanced NBS programs and support from the DOH have been essential in addressing all challenges encountered. We hope that other NBS programs in LMIC will find this information useful in seeking to improve and expand their screening capabilities.

## Figures and Tables

**Table 1:** Disorders included in the Philippine Expanded Newborn Screening Program.

Disorder Group	Disorder(s)	Abbreviation
Endocrine	Primary Congenital Hypothyroidism	CH
	Congenital Adrenal Hyperplasia (21-Hydroxylase Deficiency)	CAH
Amino Acid	Homocystinuria	HCY
	Methionine Adenosine Transferase Deficiency (Hypermethioninemia)	MAT
	Maple Syrup Urine Disease	MSUD
	Phenylketonuria	PKU
	^a^ Tyrosinemia Type I, II, III	TYR
Fatty Acid Oxidation	Carnitine Palmitoyltransferase I Deficiency	CPT1
	Carnitine Palmitoyltransferase II Deficiency	CPT2
	Carnitine Uptake Deficiency	CUD
	Glutaric Acidemia Type II	GA II
	Long Chain Hydroxyacyl-CoA Dehydrogenase Deficiency	LCHAD
	Medium Chain-Acyl-CoA Dehydrogenase Deficiency	MCAD
	Very Long Chain-Acyl-CoA Dehydrogenase Deficiency	VLCAD
	Tri-functional Protein Deficiency	TFP
Organic Acid	3-Methylcrotonyl CoA Carboxylase Deficiency	3MCC
	Beta Ketothiolase Deficiency	BKT
	Glutaric Acidemia Type I	GA1
	Isovaleric Acidemia	IVA
	Methylmalonic Acidemia	MMA
	Multiple Carboxylase Deficiency	MCD
	Propionic Acidemia	PA
Urea Cycle	Citrullinemia	CIT
	Argininosuccinic Aciduria	ASA
Hemoglobin	All Detectable Hemoglobinopathies and Thalassemias	HGB
Other	Galactosemia	GAL
	Glucose-6-Phosphate Dehydrogenase Deficiency	G6PDD
	Cystic Fibrosis	CF
	Biotinidase Deficiency	BIO

^a^ Screening methodology includes screening for both succinyl acetone and tyrosine.

**Table 2:** Metabolic disorders in Filipino newborns—California vs. Philippines (adapted from Reference [40].

Condition	^a^ Cases in Filipino Newborns Born in California 7 July 2005–6 July 2011	^a^ Prevalence in Filipino Newborns Born in California 7 July 2005–6 July 2011	^b^ Estimated Annual Cases of Filipino Newborns Born in Philippines
**Amino Acid Disorders**			
^c^ Phenylketonuria (PKU)	4	1:27,782	80
^c^ Variant Hyperphenylalaninemia	1	1:111,127	20
^c^ Maple Syrup Urine Disease (MSUD)	1	1:111,127	20
**Organic Acid Disorders**			
Methylmalonic Acidemia—MMA—(mut 0)	3	1:37,042	60
Methylmalonic Acidemia—MMA—(mut -)	2	1:55,564	40
β-Ketothiolase Deficiency (BKT)	1	1:111,127	20
Isobutyryl-CoA Dehydrogenase Deficiency (IBDHD)	1	1:111,127	20
**Fatty Acid Oxidation Disorders**			
Medium chain Acyl-CoA Dehydrogenase Deficiency (MCAD Deficiency)	2	1:55,564	40
Short Chain Acyl-CoA Dehydrogenase Deficiency (SCAD Deficiency)	3	1:37,042	60
Very Long Chain Acyl-CoA Dehydrogenase Deficiency (VLCAD deficiency)	3	1:37,042	60
Other Fatty Acid Oxidation Disorder	2	1:55,564	40
**Others**			
Partial Biotinidase Deficiency	1	1:111,127	20
CFTR-Related Metabolic Syndrome (CRMS)	5	1:22,225	100
Cystic Fibrosis	5	1:22,225	100
^c^ Classical Galactosemia	1	1:111,127	20
^c^ Duarte Galactosemia (D/G)	2	1:55,564	40
Other Disorders	2	1:55,564	40
Totals	39		780

^a^ Detected as part of California Newborn Screening Program (*n* = 111,127). Parents included: Filipino–Filipino (61,088); Filipino–White (18,546); Filipino–Hispanic (8507); Filipino–Hispanic–White (3849); Filipino–Other (19,127). ^b^ Assuming 100% coverage of 2 million annual births; overall prevalence (199 cases in 111,127 births—including 39 from conditions listed here, 109 hemoglobinopathies, and 51 endocrinopathies). ^c^ Technically this condition was already included in the Philippine NBS.

## Abbreviations

AA	Amino acids
ACMG	American College of Medical Genetics and Genomics
AO	Administrative order (Department of Health—Philippines)
ASA	Argininosuccinic aciduria
BIO	Biotinidase deficiency
CAH	Congenital adrenal hyperplasia
CDC	Centers for Disease Control and Prevention (United States)
CF	Cystic fibrosis
CH	Congenital hypothyroidism
CHGS	Center for Human Genetic Services
CNSP	California Newborn Screening Program
COVID-19	Coronavirus disease 2019
DOH	Department of Health (Philippines)
ENBS	Expanded newborn bloodspot screening
FAO	Fatty acid oxidation
G6PD	Glucose-6-phosphate dehydrogenase
GAL	Galactosemia
HCY	Homocystinuria
HGB	Hemoglobinopathy
IHG	Institute of Human Genetics (Philippines)
LIMS	Laboratory information management system
LMIC	Low- and middle-income countries
MS/MS	Tandem mass spectrometry
MSUD	Maple syrup urine disease
NBS	Newborn bloodspot screening
NBSCC	Newborn Screening Continuity Clinic (long-term follow-up)
NCNBSS	National comprehensive newborn screening system
NIH	National Institutes of Health (Philippines)
NNSGRC	National Newborn Screening and Global Resource Center (U.S.)
NSC	Newborn Screening Center (screening laboratory)
NSF	Newborn Screening Facility (screening specimen collection site)
NSQAP	Newborn Screening Quality Assurance Program (U.S.-CDC)
NSRC	Newborn Screening Reference Center (Philippines)
NTWG	Newborn Screening Technical Working Group
OA	Organic acid
PHIC	Philippine Health Insurance Corporation (national health insurance)
PKU	Phenylketonuria
PNSP	Philippine Newborn Screening Program
PT	Proficiency testing
QA	Quality assurance
QC	Quality control
RUSP	Recommended uniform screening panel (U.S.)
